# Adjunct antibody administration with standard treatment reduces relapse rates in a murine tuberculosis model of necrotic granulomas

**DOI:** 10.1371/journal.pone.0197474

**Published:** 2018-05-14

**Authors:** Alvaro A. Ordonez, Supriya Pokkali, Sunhwa Kim, Brian Carr, Mariah H. Klunk, Leah Tong, Vikram Saini, Yong S. Chang, Matthew McKevitt, Victoria Smith, David L. Gossage, Sanjay K. Jain

**Affiliations:** 1 Center for Tuberculosis Research, Johns Hopkins University School of Medicine, Baltimore, Maryland, United States of America; 2 Center for Infection and Inflammation Imaging Research, Johns Hopkins University School of Medicine, Baltimore, Maryland, United States of America; 3 Department of Pediatrics, Johns Hopkins University School of Medicine, Baltimore, Maryland, United States of America; 4 Gilead Sciences, Inc., Foster City, California, United States of America; Institut de Pharmacologie et de Biologie Structurale, FRANCE

## Abstract

Matrix metalloproteinase (MMP)-9 is a zinc-dependent protease associated with early immune responses to *Mycobacterium tuberculosis* infection, macrophage recruitment and granuloma formation. We evaluated whether adjunctive inhibition of MMP-9 could improve the response to standard TB treatment in a mouse model that develops necrotic lesions. Six weeks after an aerosol infection with *M*. *tuberculosis*, C3HeB/FeJ mice received standard TB treatment (12 weeks) comprising rifampin, isoniazid and pyrazinamide alone or in combination with either anti-MMP-9 antibody, etanercept (positive control) or isotype antibody (negative control) for 6 weeks. Anti-MMP-9 and the isotype control had comparable high serum exposures and expected terminal half-life. The relapse rate in mice receiving standard TB treatment was 46.6%. Compared to the standard TB treatment, relapse rates in animals that received adjunctive treatments with anti-MMP-9 antibody or etanercept were significantly decreased to 25.9% (*P* = 0.006) and 29.8% (*P* = 0.019) respectively, but were not different from the arm that received the isotype control antibody (25.9%). Immunostaining demonstrated localization of MMP-9 primarily in macrophages in both murine and human lung tissues infected with *M*. *tuberculosis*, suggesting the importance of MMP-9 in TB pathogenesis. These data suggest that the relapse rates in *M*. *tuberculosis*-infected mice may be non-specifically improved by administration of antibodies in conjunction with standard TB treatments. Future studies are needed to evaluate the mechanism(s) leading to improved outcomes with adjunctive antibody treatments.

## Introduction

*Mycobacterium tuberculosis* is a global threat and a major cause of human morbidity and mortality [1]. The development of multi-drug resistant strains and the limited arsenal of effective tuberculosis (TB) treatment regimens, which need to be administered for prolonged periods (6 months for fully drug susceptible TB), are major challenge to controlling TB leading to an urgent need for shorter regimens. Most of the current components of TB treatments target actively replicating bacteria and thus require protracted treatments to eliminate the slow-replicating “persistent” bacteria and achieve stable cure. Moreover, it is possible that the early host-responses to *M*. *tuberculosis*, i.e., necrosis, hypoxia, granuloma formation and cavitation, could be detrimental to bacterial clearance [2].

Matrix metalloproteinases (MMPs) are a family of zinc-dependent proteases known to degrade collagen and remodel the extracellular matrix and basement membranes [3]. Multiple MMPs have been associated with TB pathogenesis, including MMP-9, an extracellular gelatinase that specifically degrades type IV and V collagen [4–7]. In humans with pulmonary, pleural or CNS TB, increased MMP-9 activity in serum and CSF correlates with increased disease severity and worse outcomes [8–10]. Antisense knock down of MMP-9 in zebrafish infected with *Mycobacterium marinum* attenuated granuloma formation and bacterial growth [11]. In *M*. *tuberculosis*-infected mice, disruption of MMP-9 expression or activity reduces macrophage infiltration into the lungs, leading to the dysregulation of granuloma formation and reduced disease burden [12]. Therefore, inhibiting MMP-9 expression or activity could disrupt TB-associated granulomas thereby enhancing the activity of antimicrobials against persistent bacteria. Here, we evaluated an inhibitory antibody against MMP-9 [13] as adjunctive treatment in combination with standard TB treatments in *M*. *tuberculosis*-infected C3HeB/FeJ mice, which develop well-organized, hypoxic TB granulomas as well as cavitary lesions after aerosol infection [7, 14, 15].

## Materials and methods

All procedures were approved by the ethics and Animal Care and Use committees of Johns Hopkins University. All experiments with *M*. *tuberculosis* were performed according to biosafety procedures in the animal biological safety level-3 (ABSL-3) facility at Johns Hopkins University.

### Animal experiments

#### *In vivo* aerosol infection

The experimental scheme is outlined in S1 Fig. Four to six-week-old female C3HeB/FeJ (Jackson Laboratory) mice were aerosol infected with frozen titrated bacterial stocks of *M*. *tuberculosis* H37Rv [5.66 ± 0.13 log_10_ colony-forming units (CFU)/ml], using the Middlebrook Inhalation Exposure System (Glas-Col). The animals were housed inside the ABSL-3 facility with water and food available *ad libitum* and monitored daily for signs of distress after infection. Mice were sacrificed using isoflurane (Henry Schein) overdose one day after infection (to assess implantation) and at the start of treatment at six weeks after infection. Mice were also sacrificed 2, 6, 8, 10 and 12 weeks after starting TB treatments. A minimum of seven mice were used per group and for each time-point. Whole lungs were removed aseptically, homogenized in PBS and plated by serial dilution in duplicate, onto Middlebrook 7H11 agar plates (Becton Dickinson). Plates were incubated at 37°C for 4 weeks before CFU were counted.

#### Multi-drug TB treatments

Standard TB treatment entailed the administration of rifampin (R, 10mg/kg/day), isoniazid (H, 10mg/kg/day) and pyrazinamide (Z, 150mg/kg/day) by gavage, 5 days per week for a total of 12 weeks as described previously [16]. Pyrazinamide was administered for the first 8 weeks only, as is standard for TB treatment in humans [17]. The mice were split among five treatment groups: no treatment, standard TB treatment (RHZ), standard TB treatment with 15mg/kg etanercept (Amgen) injected intraperitoneally twice weekly (positive control) [16], standard TB treatment with isotype control antibody (AB5123, Gilead Sciences, Inc.) (negative control), and standard TB treatment with antibody against MMP-9 (AB0046, Gilead Sciences, Inc.) for 6 weeks. Both AB0046 and AB5123 were administered at 20 mg/kg intraperitoneally, twice weekly after a single injection of 50 mg/kg at the start of treatment as a loading dose.

#### Relapse

To assess for stable, relapse free cure, additional cohorts of mice were held for 16 weeks after cessation of treatment. At this time, lungs and spleens were removed aseptically, homogenized and plated on Middlebrook 7H11 agar plates. The complete homogenate for each organ was plated across several plates.

#### Serum antibody concentrations

An indirect ELISA was utilized for the quantitation of anti-MMP-9 (AB0046) and isotype control (AB5123) in mouse serum. Briefly, standard 96-well microtiter plates were coated with mouse MMP-9 (R&D Systems) and CHOsAg (ProSpec) to capture AB0046 and AB5123, respectively. AB0046 and AB5123 in mouse serum were detected using goat anti-mouse IgG horseradish peroxidase conjugate (HRP) followed by incubation with 3,3’,5,5’-tetramethylbenzidine (TMB) (Sigma) substrate solution to produce a color reaction stopped by the addition of 1 M HCl. The absorbance was measured at 450 nm using a standard plate reader. The intensity of the color produced is proportional to the amount of AB0046 and AB5123 present in the serum sample extrapolated against a standard curve established with AB0046 and AB5123, respectively, using a 4-parameter logistic curve fit.

#### Histopathology

Lungs were harvested after systemic perfusion with phosphate-buffered saline under deep anesthesia, fixed in 4% paraformaldehyde and sectioned to 5μm thickness. Hematoxylin-eosin, acid-fast and Masson's trichrome staining was performed as described previously [7, 18]. Images shown are representative of sections obtained from a minimum of four animals per group for each time-point.

#### Immunohistochemistry

Paraffin embedded sections were rehydrated in graded alcohols, steamed in citrate buffer at pH 6 and probed at room temperature for 2 hours using the MMP-9 (rabbit polyclonal; 1:250; Abcam [AB38898]) and processed with a polymer-HRP kit (BioGenex) with diaminobenzidine development and Mayer hematoxylin counterstaining. Lungs from uninfected, and infected but untreated animals without primary antibody served as negative controls. Slides were scanned using the Apeiro digital scanner (Leica). For human tissue immunohistochemistry, de-identified, post mortem samples from patients with pulmonary TB were obtained from the Johns Hopkins Hospital Department of Pathology collection and processed similarly for immunohistochemistry with the human anti-MMP-9 antibody (AB76003, rabbit monoclonal; 1:250; Abcam [EP1254]).

#### Computed tomography imaging

A subset of live *M*. *tuberculosis*-infected animals from the relapse cohort were imaged within a sealed bio-containment bed (Minerve) modified in-house to be compliant with biosafety level 3 (BSL3) containment, as described previously [7, 19]. A standard small animal anesthesia machine was used to deliver a mixture of isoflurane and oxygen to anesthetize during imaging. Computed tomography (CT) scans were then immediately performed using the NanoSPECT/CT (Mediso) *in vivo* animal imager. The images were analyzed using VivoQuant 2.5 (Invicro). The whole lung field was segmented and the frequency of voxels with a given density (measured as Hounsfield Units, HU) was determined. A cavity was defined as a macroscopic region of air (density <−900 HU) within diseased lung parenchyma.

#### Statistical analysis

Statistical comparison between groups was performed using a two-tailed Fisher’s Exact or a two-tailed Mann-Whitney test in Prism 6 version 6.07 (GraphPad). Data are presented on a logarithmic scale as mean ± standard deviation (SD) for CFU counts, except where stated.

## Results

After a low-dose aerosol infection with *M*. *tuberculosis*, the pulmonary bacterial implantation in mice was 1.60 ± 0.39 log_10_ CFU. Six weeks after the infection when TB treatments were initiated the pulmonary bacterial burden was 6.87 ± 0.11 log_10_ CFU.

### MMP-9 expression in *M*. *tuberculosis*-infected tissues

**Fig 1:**
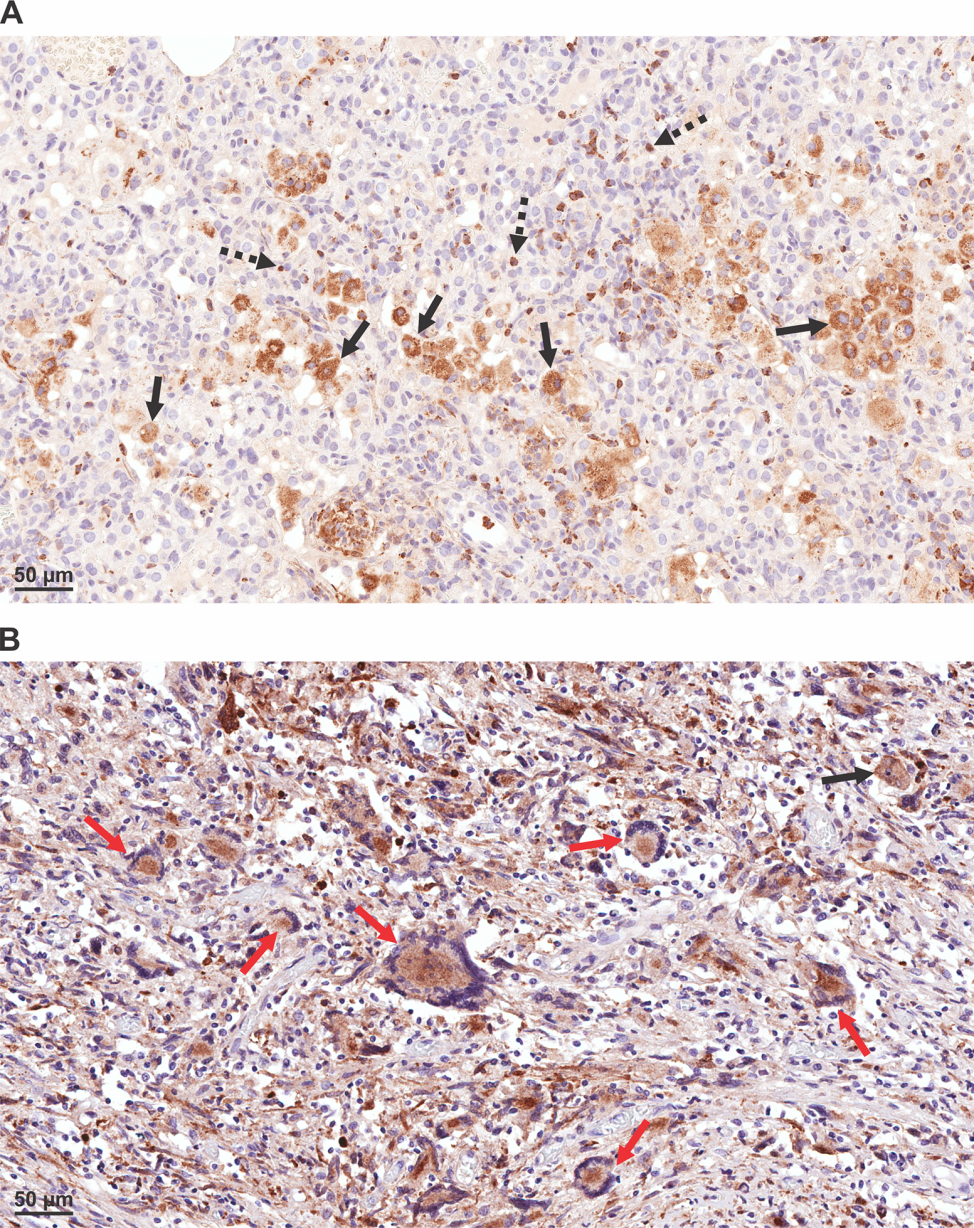
MMP-9 expression in mouse and human *M*. *tuberculosis*-infected tissues. (A) MMP-9 immunohistochemistry in the lung tissue of a C3HeB/FeJ mouse, 10 weeks after *M*. *tuberculosis* aerosol infection. MMP-9 was visualized in macrophages (black solid arrows) and neutrophils (black dotted arrows). (B) Expression of MMP-9 in the human lung tissue with cavitary TB. MMP-9 was expressed in macrophages (black arrow) and multinucleated giant cells (red arrows).

To evaluate the expression of MMP-9 after *M*. *tuberculosis* infection we qualitatively assessed MMP-9 using immunohistochemistry in mouse and human lung tissues. Immunostaining demonstrated localization of MMP-9 mostly with macrophages in both human and mouse tissues (Fig 1). However, in mouse lungs there was also an increased MMP-9 signal associated with neutrophils.

### Serum antibody concentrations

**Fig 2:**
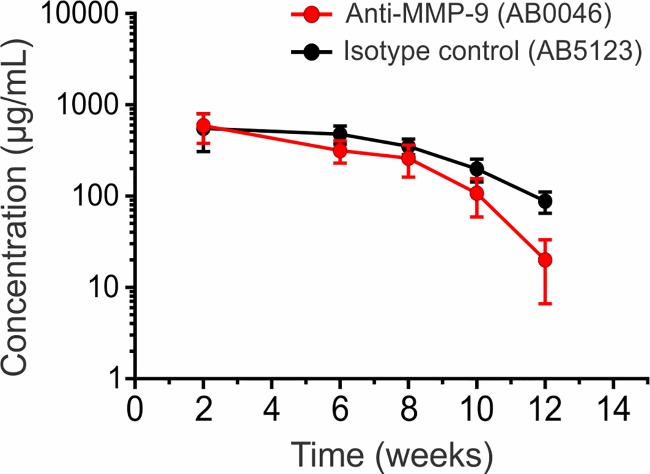
Serum concentrations of anti-MMP-9 and isotype control antibodies in mice. Mean serum concentrations (± SD) from individual mice receiving standard TB treatment in combination with anti-MMP-9 (AB0046, red circle) or isotype control (AB5123, black circle) are depicted. Mice were sacrificed at week 2 (n = 13) and 6 (n = 13) during the dosing phase, and at week 8 (n = 13), 10 (n = 7), and 12 (n = 7) during the antibody elimination phase.

Anti-MMP-9 (AB0046) and isotype control (AB5123) had comparable high serum exposures, ranging from approximately 300–600 μg/mL by 6 weeks of dosing. After dosing, the antibodies displayed the expected terminal half-lives based on historical data, with AB0046 showing faster elimination due to target mediated disposition (Fig 2). No significant apparent immunogenicity was noted in the animals based on aberrant declines in antibody titers during dosing and antibody elimination.

### Adjunctive treatments improve bacterial clearance

**Fig 3:**
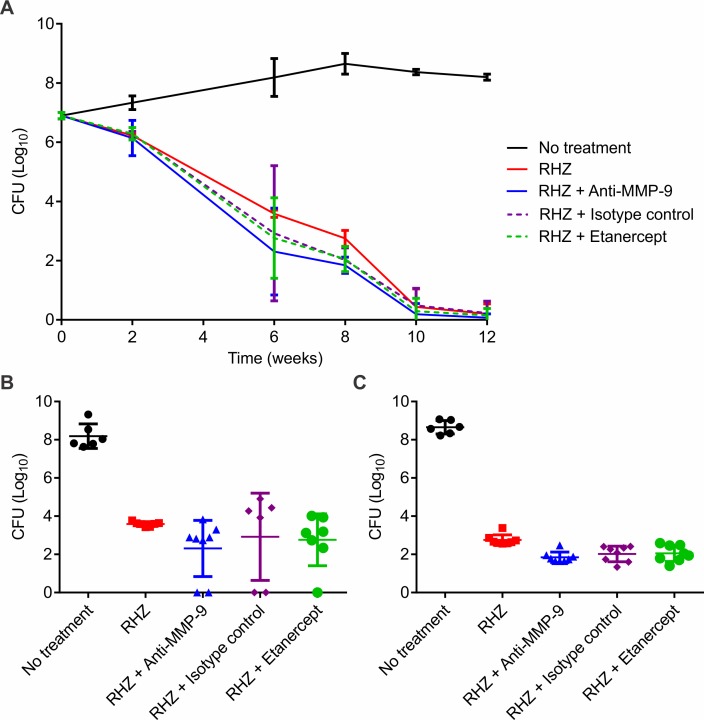
Bacterial burden in the lungs of mice. Six weeks after an aerosol infection with *Mycobacterium tuberculosis*, C3HeB/FeJ mice were split among several treatment arms and the number of viable bacteria in the lungs were estimated by determining colony-forming units (CFU). Results are shown for the duration of study (A) and also as individual dot plots for 6 (B) and 8 (C) weeks after starting TB treatment. Results are presented as mean (±SD) CFU in the lungs, detected from a minimum of seven mice at each time point and for each group. CFU are presented on a logarithmic scale (log_10_). RHZ = standard TB treatment comprising rifampin (R), isoniazid (H) and pyrazinamide (Z) administered by gavage. Pyrazinamide was administered for the first 8 weeks only.

The addition of adjunctive treatments did not modify the bacterial burden during the initial phase of TB treatment, when most of the bacteria are actively replicating (Fig 3A). However, at later time-points (6 and 8 weeks of treatment), when a significant proportion of bacteria are dividing slowly (persisters), the addition of adjunctive treatments resulted in a significantly lower bacterial burden compared to the standard treatment alone (*P* = 0.012 and <0.001 at 6 and 8 weeks, respectively) with a trend towards better outcome in the arm receiving anti-MMP-9 treatment (Fig 3B and 3C). Post-mortem gross pathological lungs samples at 6 (panel A) and 8 (panel B) weeks of treatment are shown in S2 Fig.

**Fig 4:**
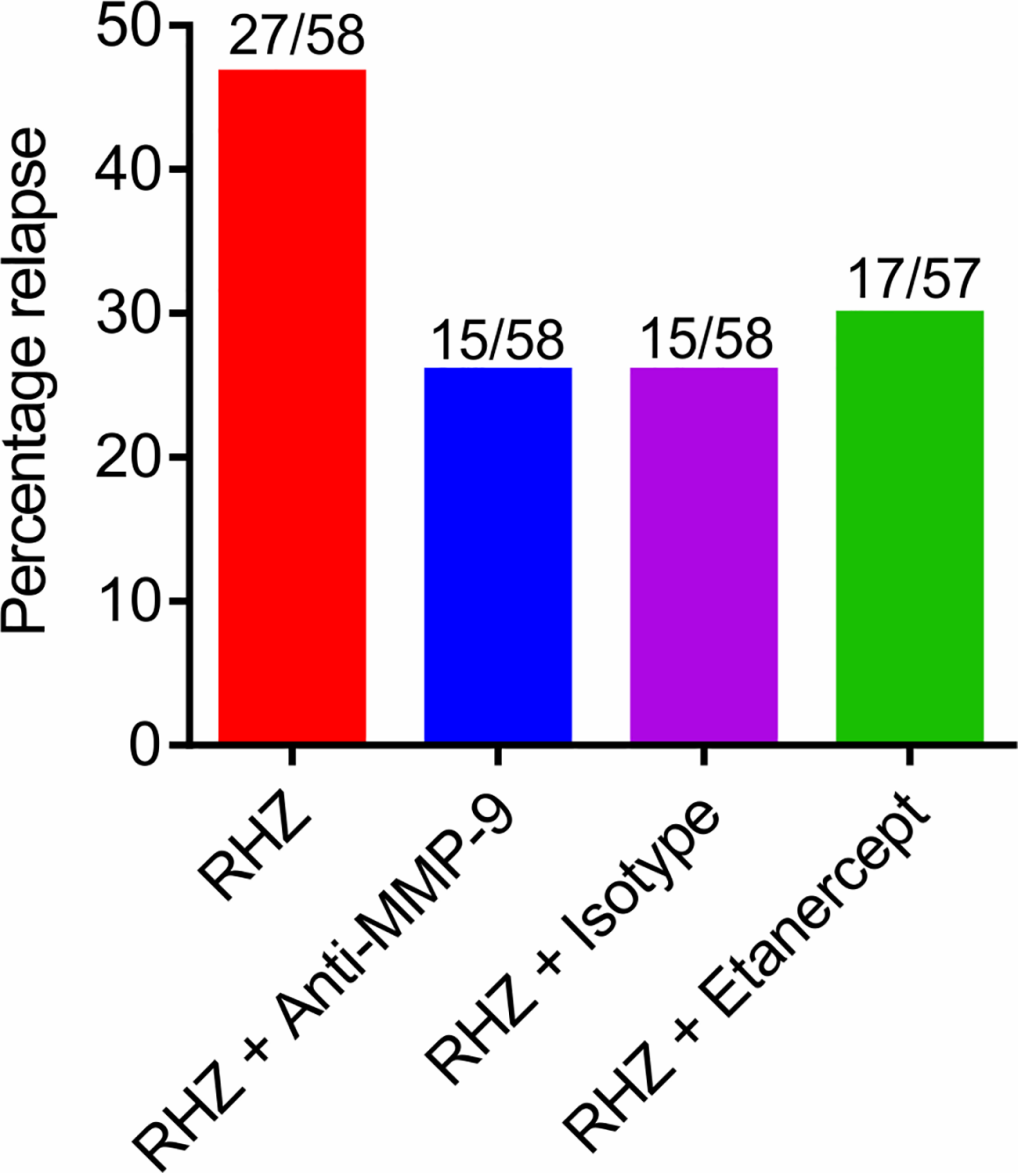
Relapse rates associated with each treatment arm. Additional cohorts of mice were held for 16 weeks after cessation of treatment to assess for stable, relapse free cure. Results are presented as proportion of mice with any viable bacteria in the lungs. RHZ = standard TB treatment comprising rifampin (R), isoniazid (H) and pyrazinamide (Z) administered by gavage.

Additional cohorts of mice were held for 16 weeks after cessation of treatment to assess for stable cure (Fig 4). The relapse rate in mice that were administered standard TB treatment for 12 weeks was 46.6% (27 of 58). Consistent with our prior data [17], adjunctive use of etanercept significantly reduced the relapse rates to 29.8% (17 of 57; *P* = 0.019). Animals that received adjunctive treatments with anti-MMP-9 also had a significantly lower relapse rate 25.9% (15 of 58; *P* = 0.006) but no different from the arm that received the isotype control antibody (25.9%; 15 of 58; *P* = 1.000). There were no significant differences between the groups in the number of cavities as evaluated by CT (S3 Fig).

In summary, we evaluated an inhibitory antibody against MMP-9 as adjunctive treatment in combination with standard TB treatments in *M*. *tuberculosis*-infected C3HeB/FeJ mice, which develop well-organized, hypoxic TB granulomas as well as cavitary lesions after aerosol infection. After 6 weeks of treatment, the addition of adjunctive treatments significantly reduced the bacterial burden compared to the standard treatment alone. During the relapse phase, animals that received adjunctive treatments with antibody against MMP-9 as well as the control isotype antibody had a significantly lower relapse rate.

## Discussion

While current TB treatments are highly effective in targeting actively replicating bacteria, killing the slow-replicating, persistent microbes requires extended treatment with multiple drugs. Early host-responses (inflammation, necrosis and subsequent hypoxia) may promote survival of persistent bacteria and limit the effectiveness of TB treatments [20]. Therefore, adjuvant host-directed therapies that modulate the immune response may be beneficial during early phases of TB treatments [21]. There is strong evidence based on randomized controlled trials that adjunctive use of corticosteroid for the initial 6–8 weeks improves survival in HIV-negative patients with TB meningitis [22]. However, the value of host-directed treatments is not well established for other forms of TB, though some studies suggest there are some benefits [23–25]. For example, tumor necrosis factor alpha (TNF-α) has a key role in the host response against TB[26], and adjunctive use of TNF-α inhibitors during TB treatments may paradoxically be beneficial [16, 27–30]. TNF-α levels increase shortly after initiation of TB treatment [31], causing tissue destruction, which could favor bacterial survival, and thus inhibition of TNF-α or other such pro-inflammatory pathways may be useful during the early phase of TB treatment.

We have previously shown MMP-9 expression in necrotic and cavitary lesions of *M*. *tuberculosis*-infected mice, with a corresponding decrease during TB treatment [7]. Therefore, in the current study we evaluated MMP-9 expression (utilizing immunohistochemistry) in *M*. *tuberculosis*-infected murine tissues as well as post-mortem specimens from TB patients. While MMP-9 was predominantly associated with macrophage lineage in human lung tissues, MMP-9 expression was also noted in neutrophils in murine lung tissues. It should be noted that we utilized species-specific antibodies for immunohistochemistry—AB38898 directed against murine MMP-9 and AB76003 directed against human MMP-9. However, since these antibodies target different epitopes in murine and human MMP-9, differential staining patterns could be observed in murine and human tissues. Moreover, given that MMP expression and distribution is likely to be species specific [32, 33], these patterns could also be attributed to biological differences between the two mammalian species.

MMP-9 has been implicated in the pathogenesis of several diseases including TB [8–10], and selective inhibition of MMP-9 (using AB0046) was efficacious in preclinical models of ulcerative colitis and colorectal cancer [13]. We therefore evaluated whether inhibition of MMP-9 during the early phase of TB treatments in mice with established granulomatous pulmonary TB, a situation analogous to human patients presenting with active TB, could hasten bacterial clearance and provide stable cure. The anti-MMP9 and the isotype control arms had relatively high serum exposures in mice and no significant immunogenicity was noted. Importantly and as expected, the addition of adjunctive treatments did not alter bactericidal activity during the initial phase of TB treatment. However, at later time-points (6 and 8 weeks of treatment), the addition of adjunctive treatments resulted in a significantly lower bacterial burden as compared to the standard treatment alone with a trend towards a better outcome in the arm receiving anti-MMP-9.

Additional cohorts of mice were held for 16 weeks after cessation of treatment to assess for stable (relapse-free) cure. Consistent with our prior data, adjunctive use of etanercept (positive control) significantly reduced the relapse rates compared to the standard TB treatment arm [16]. Similarly, animals that received adjunctive treatments with anti-MMP-9 had a significantly lower relapse rate of 25.9% (versus 46.6% for the standard treatment arm), but it was no different from the animals that received the isotype control antibody. This was an unexpected finding. Non-specific polyclonal antibodies, such as intravenous immunoglobulins (IVIG) have shown some protective effects in mouse models of *M*. *tuberculosis* [34–37]. There is also evidence for the potential of antibody-based treatments in improving TB treatments [38, 39], with a faster reduction of the pulmonary bacillary burden when IVIG was administered in combination with standard TB treatment [40]. While the exact mechanism(s) of action are not entirely known, it is possible that both the neutralization of pathogens as well as the immune-modulatory activities may be contributing to this effect [40–42]. However, given that none of the adjunctive antibody treatments utilized in the current study were specific for mycobacterial antigens, this effect is most likely mediated by a non-specific ability of the antibodies to modulate inflammation. The protective effect of IVIG in TB has been suggested to be dependent on antibody binding to the fragment crystallizable region receptor (FcR), which significantly increased phagocytosis and intracellular killing of *M*. *tuberculosis* by macrophages when exposed to IVIG *in vitro* [37, 40]. Furthermore, the presence of sialylated glycans in the Fc-portion of antibodies increases binding affinity to the FcR, leading to increased antibody-dependent cellular cytotoxicity [43], which could have an effect on the survival of the mycobacteria.

In summary, we evaluated the potential of adjunctive MMP-9 inhibition in combination with standard TB treatments in a murine model that develop well-organized hypoxic TB granulomas and cavitary lesions. Animals that received adjunctive treatments with anti-MMP-9 antibody had a significantly lower relapse rate but it was no different from the arm that received the isotype control antibody. Given that none of the adjunctive treatments utilized in the current study were specific for mycobacterial antigens, these improved treatments are likely a host-directed effect. Future studies will evaluate the mechanism(s) for this outcome.

## Supporting information

S1 Fig Experimental scheme. Four to six-week-old female C3HeB/FeJ mice were aerosol infected with *Mycobacterium tuberculosis*. Mice were sacrificed one day after infection (to assess implantation) and at the start of treatment at six weeks after infection. Mice received multi-drug TB treatments with and without adjunctive treatments for 12 weeks and were split among six treatment groups: no treatment, standard TB treatment (administered rifampin, isoniazid and pyrazinamide by gavage), or standard TB treatment with either adjunctive etanercept (positive control), isotype antibody (negative control) or anti-MMP-9 antibody for 6 weeks. Pyrazinamide was administered for the first 8 weeks only, as is standard for TB treatment in humans. Additional cohorts of mice were held for 16 weeks after cessation of treatment to assess for stable, relapse free cure.
(DOCX)

S2 Fig Post-mortem gross pathological lungs samples. Mice were sacrificed at 6 (A) and 8 (B) weeks of treatment and the lungs were harvested, fixed in 4% paraformaldehyde and gross images were acquired. RHZ = standard TB treatment comprising rifampin (R), isoniazid (H) and pyrazinamide (Z) administered by gavage.
(DOCX)

S3 Fig Presence of cavities in each treatment arm during relapse. Additional cohorts of mice were held for 16 weeks after cessation of treatment to assess for stable, relapse free cure. 20 animals per group were CT scanned to evaluate for the presence of cavitary lesions. The transverse, coronal and sagittal views of a CT from a representative mouse scanned 16 weeks post-treatment are shown in panel A. The crosshairs indicate the cavitary lesion. A cavity was defined as a macroscopic region of air (density <−900 HU) within diseased lung parenchyma. (B) The number of cavitary lesions was quantified by CT in each of the scanned mice within the relapse groups. RHZ = standard TB treatment comprising rifampin (R), isoniazid (H) and pyrazinamide (Z) administered by gavage.
(DOCX)

## References

[1] WHO. Global TB Report 2017 [April 1, 2018]. Available from: http://www.who.int/tb/publications/global_report/en/.

[2] Paige C, Bishai WR. Penitentiary or penthouse condo: the tuberculous granuloma from the microbe's point of view. Cell Microbiol. 2010;12(3):301–9. doi: 10.1111/j.1462-5822.2009.01424.x . 20039878 10.1111/j.1462-5822.2009.01424.x

[3] Greenlee KJ, Werb Z, Kheradmand F. Matrix metalloproteinases in lung: multiple, multifarious, and multifaceted. Physiol Rev. 2007;87(1):69–98. doi: 10.1152/physrev.00022.2006 ; PubMed Central PMCID: PMCPMC2656382. 17237343 10.1152/physrev.00022.2006 PMC2656382

[4] Salgame P. MMPs in tuberculosis: granuloma creators and tissue destroyers. The Journal of clinical investigation. 2011;121(5):1686–8. doi: 10.1172/JCI57423 ; PubMed Central PMCID: PMC3083780. 21519148 10.1172/JCI57423 PMC3083780

[5] Ong CW, Elkington PT, Friedland JS. Tuberculosis, pulmonary cavitation, and matrix metalloproteinases. American journal of respiratory and critical care medicine. 2014;190(1):9–18. doi: 10.1164/rccm.201311-2106PP ; PubMed Central PMCID: PMC4226026. 24713029 10.1164/rccm.201311-2106PP PMC4226026

[6] Kubler A, Luna B, Larsson C, Ammerman NC, Andrade BB, Orandle M, et al Mycobacterium tuberculosis dysregulates MMP/TIMP balance to drive rapid cavitation and unrestrained bacterial proliferation. The Journal of pathology. 2015;235(3):431–44. doi: 10.1002/path.4432 ; PubMed Central PMCID: PMC4293239. 25186281 10.1002/path.4432 PMC4293239

[7] Ordonez AA, Tasneen R, Pokkali S, Xu Z, Converse PJ, Klunk MH, et al Mouse model of pulmonary cavitary tuberculosis and expression of matrix metalloproteinase-9. Dis Model Mech. 2016;9(7):779–88. doi: 10.1242/dmm.025643 ; PubMed Central PMCID: PMCPMC4958312. 27482816 10.1242/dmm.025643 PMC4958312

[8] Price NM, Farrar J, Chau TTH, Mai NTH, Hien TT, Friedland JS. Identification of a matrix-degrading phenotype in human tuberculosis in vitro and in vivo. The Journal of Immunology. 2001;166(6):4223–30. 11238675 10.4049/jimmunol.166.6.4223

[9] Hrabec E, Strek M, Zieba M, Kwiatkowska S, Hrabec Z. Circulation level of matrix metalloproteinase-9 is correlated with disease severity in tuberculosis patients. The International Journal of Tuberculosis and Lung Disease. 2002;6(8):713–9. 12150484

[10] Sheen P, O’Kane CM, Chaudhary K, Tovar M, Santillan C, Sosa J, et al High MMP-9 activity characterises pleural tuberculosis correlating with granuloma formation. European Respiratory Journal. 2009;33(1):134–41. doi: 10.1183/09031936.00127807 18715875 10.1183/09031936.00127807

[11] Volkman HE, Pozos TC, Zheng J, Davis JM, Rawls JF, Ramakrishnan L. Tuberculous granuloma induction via interaction of a bacterial secreted protein with host epithelium. Science. 2010;327(5964):466–9. doi: 10.1126/science.1179663 ; PubMed Central PMCID: PMCPMC3125975. 20007864 10.1126/science.1179663 PMC3125975

[12] Taylor JL, Hattle JM, Dreitz SA, Troudt JM, Izzo LS, Basaraba RJ, et al Role for matrix metalloproteinase 9 in granuloma formation during pulmonary Mycobacterium tuberculosis infection. Infect Immun. 2006;74(11):6135–44. doi: 10.1128/IAI.02048-05 ; PubMed Central PMCID: PMCPMC1695484. 16982845 10.1128/IAI.02048-05 PMC1695484

[13] Marshall DC, Lyman SK, McCauley S, Kovalenko M, Spangler R, Liu C, et al Selective Allosteric Inhibition of MMP9 Is Efficacious in Preclinical Models of Ulcerative Colitis and Colorectal Cancer. PLoS One. 2015;10(5):e0127063 doi: 10.1371/journal.pone.0127063 ; PubMed Central PMCID: PMCPMC4427291. 25961845 10.1371/journal.pone.0127063 PMC4427291

[14] Harper J, Skerry C, Davis SL, Tasneen R, Weir M, Kramnik I, et al Mouse Model of Necrotic Tuberculosis Granulomas Develops Hypoxic Lesions. Journal of Infectious Diseases. 2012;205(4):595–602. doi: 10.1093/infdis/jir786 22198962 10.1093/infdis/jir786 PMC3266133

[15] Pan H, Yan BS, Rojas M, Shebzukhov YV, Zhou H, Kobzik L, et al Ipr1 gene mediates innate immunity to tuberculosis. Nature. 2005;434(7034):767–72. Epub 2005/04/09. doi: 10.1038/nature03419 ; PubMed Central PMCID: PMCPMC1388092. 15815631 10.1038/nature03419 PMC1388092

[16] Skerry C, Harper J, Klunk M, Bishai WR, Jain SK. Adjunctive TNF inhibition with standard treatment enhances bacterial clearance in a murine model of necrotic TB granulomas. PLoS One. 2012;7(6):e39680 doi: 10.1371/journal.pone.0039680 ; PubMed Central PMCID: PMCPMC3384606. 22761866 10.1371/journal.pone.0039680 PMC3384606

[17] Blumberg HM, Burman WJ, Chaisson RE, Daley CL, Etkind SC, Friedman LN, et al American Thoracic Society/Centers for Disease Control and Prevention/Infectious Diseases Society of America: treatment of tuberculosis. American journal of respiratory and critical care medicine. 2003;167(4):603–62. doi: 10.1164/rccm.167.4.603 . 12588714 10.1164/rccm.167.4.603

[18] Ordonez AA, Pokkali S, DeMarco VP, Klunk M, Mease RC, Foss CA, et al Radioiodinated DPA-713 imaging correlates with bactericidal activity of tuberculosis treatments in mice. Antimicrobial agents and chemotherapy. 2015;59(1):642–9. doi: 10.1128/AAC.04180-14 ; PubMed Central PMCID: PMC4291409. 25403669 10.1128/AAC.04180-14 PMC4291409

[19] Davis SL, Nuermberger EL, Um PK, Vidal C, Jedynak B, Pomper MG, et al Noninvasive pulmonary [18F]-2-fluoro-deoxy-D-glucose positron emission tomography correlates with bactericidal activity of tuberculosis drug treatment. Antimicrobial agents and chemotherapy. 2009;53(11):4879–84. doi: 10.1128/AAC.00789-09 ; PubMed Central PMCID: PMC2772305. 19738022 10.1128/AAC.00789-09 PMC2772305

[20] Collins HL, Kaufmann SH. The many faces of host responses to tuberculosis. Immunology. 2001;103(1):1–9. doi: 10.1046/j.1365-2567.2001.01236.x 11380686 10.1046/j.1365-2567.2001.01236.x PMC1783212

[21] Ordonez AA, Maiga M, Gupta S, Weinstein EA, Bishai WR, Jain SK. Novel adjunctive therapies for the treatment of tuberculosis. Current molecular medicine. 2014;14(3):385–95. 24236454 10.2174/1566524013666131118112431 PMC4484774

[22] Thwaites GE, Nguyen DB, Nguyen HD, Hoang TQ, Do TT, Nguyen TC, et al Dexamethasone for the treatment of tuberculous meningitis in adolescents and adults. N Engl J Med. 2004;351(17):1741–51. doi: 10.1056/NEJMoa040573 . 15496623 10.1056/NEJMoa040573

[23] Kaufmann SH, Lange C, Rao M, Balaji KN, Lotze M, Schito M, et al Progress in tuberculosis vaccine development and host-directed therapies—a state of the art review. The Lancet Respiratory Medicine. 2014;2(4):301–20. doi: 10.1016/S2213-2600(14)70033-5 24717627 10.1016/S2213-2600(14)70033-5

[24] Dorhoi A, Kaufmann SH. Perspectives on host adaptation in response to Mycobacterium tuberculosis: modulation of inflammation. Semin Immunol. 2014;26(6):533–42. Epub 2014/12/03. doi: 10.1016/j.smim.2014.10.002 . 25453228 10.1016/j.smim.2014.10.002

[25] Muthuswamy P, Hu TC, Carasso B, Antonio M, Dandamudi N. Prednisone as adjunctive therapy in the management of pulmonary tuberculosis. Report of 12 cases and review of the literature. Chest. 1995;107(6):1621–30. Epub 1995/06/01. . 10.1378/chest.107.6.1621 7781357

[26] Flynn JL, Goldstein MM, Chan J, Triebold KJ, Pfeffer K, Lowenstein CJ, et al Tumor necrosis factor-alpha is required in the protective immune response against Mycobacterium tuberculosis in mice. Immunity. 1995;2(6):561–72. . 7540941 10.1016/1074-7613(95)90001-2

[27] Wallis RS, van Vuuren C, Potgieter S. Adalimumab treatment of life-threatening tuberculosis. Clin Infect Dis. 2009;48(10):1429–32. Epub 2009/04/15. doi: 10.1086/598504 . 19364287 10.1086/598504

[28] Wallis RS. Reconsidering adjuvant immunotherapy for tuberculosis. Clin Infect Dis. 2005;41(2):201–8. Epub 2005/06/29. doi: CID35799 [pii] doi: 10.1086/430914 . 15983916 10.1086/430914

[29] Blackmore TK, Manning L, Taylor WJ, Wallis RS. Therapeutic use of infliximab in tuberculosis to control severe paradoxical reaction of the brain and lymph nodes. Clin Infect Dis. 2008;47(10):e83–5. Epub 2008/10/09. doi: 10.1086/592695 . 18840076 10.1086/592695

[30] Wallis RS, Kyambadde P, Johnson JL, Horter L, Kittle R, Pohle M, et al A study of the safety, immunology, virology, and microbiology of adjunctive etanercept in HIV-1-associated tuberculosis. AIDS. 2004;18(2):257–64. 15075543 10.1097/00002030-200401230-00015

[31] Bekker LG, Maartens G, Steyn L, Kaplan G. Selective increase in plasma tumor necrosis factor-alpha and concomitant clinical deterioration after initiating therapy in patients with severe tuberculosis. J Infect Dis. 1998;178(2):580–4. Epub 1998/08/11. . 9697749 10.1086/517479

[32] Singh S, Kubler A, Singh UK, Singh A, Gardiner H, Prasad R, et al Antimycobacterial drugs modulate immunopathogenic matrix metalloproteinases in a cellular model of pulmonary tuberculosis. Antimicrobial agents and chemotherapy. 2014;58(8):4657–65. doi: 10.1128/AAC.02141-13 ; PubMed Central PMCID: PMC4136059. 24890593 10.1128/AAC.02141-13 PMC4136059

[33] Elkington P, Shiomi T, Breen R, Nuttall RK, Ugarte-Gil CA, Walker NF, et al MMP-1 drives immunopathology in human tuberculosis and transgenic mice. The Journal of clinical investigation. 2011;121(5):1827–33. doi: 10.1172/JCI45666 ; PubMed Central PMCID: PMCPMC3083790. 21519144 10.1172/JCI45666 PMC3083790

[34] Roy E, Stavropoulos E, Brennan J, Coade S, Grigorieva E, Walker B, et al Therapeutic efficacy of high-dose intravenous immunoglobulin in Mycobacterium tuberculosis infection in mice. Infect Immun. 2005;73(9):6101–9. Epub 2005/08/23. doi: 10.1128/IAI.73.9.6101-6109.2005 ; PubMed Central PMCID: PMCPMC1231090. 16113331 10.1128/IAI.73.9.6101-6109.2005 PMC1231090

[35] Alvarez N, Otero O, Camacho F, Borrero R, Tirado Y, Puig A, et al Passive administration of purified secretory IgA from human colostrum induces protection against Mycobacterium tuberculosis in a murine model of progressive pulmonary infection. BMC Immunol. 2013;14 Suppl 1:S3 Epub 2013/03/15. doi: 10.1186/1471-2172-14-S1-S3 ; PubMed Central PMCID: PMCPMC3582447. 23458564 10.1186/1471-2172-14-S1-S3 PMC3582447

[36] Olivares N, Puig A, Aguilar D, Moya A, Cadiz A, Otero O, et al Prophylactic effect of administration of human gamma globulins in a mouse model of tuberculosis. Tuberculosis (Edinb). 2009;89(3):218–20. Epub 2009/04/14. doi: 10.1016/j.tube.2009.02.003 . 19362883 10.1016/j.tube.2009.02.003

[37] Olivares N, Marquina B, Mata-Espinoza D, Zatarain-Barron ZL, Pinzon CE, Estrada I, et al The protective effect of immunoglobulin in murine tuberculosis is dependent on IgG glycosylation. Pathog Dis. 2013;69(3):176–83. doi: 10.1111/2049-632X.12069 . 23873753 10.1111/2049-632X.12069

[38] Achkar JM, Casadevall A. Antibody-mediated immunity against tuberculosis: implications for vaccine development. Cell Host Microbe. 2013;13(3):250–62. doi: 10.1016/j.chom.2013.02.009 ; PubMed Central PMCID: PMCPMC3759397. 23498951 10.1016/j.chom.2013.02.009 PMC3759397

[39] Reljic R, Ivanyi J. Immunotherapy of tuberculosis with IgA and cytokines Understanding Tuberculosis-Analyzing the Origin of Mycobacterium Tuberculosis Pathogenicity: InTech; 2012.

[40] Olivares N, Rodriguez Y, Zatarain-Barron ZL, Marquina B, Mata-Espinosa D, Barrios-Payan J, et al A significant therapeutic effect of immunoglobulins administered alone, or in combination with conventional chemotherapy, in experimental pulmonary tuberculosis caused by drug-sensitive or drug-resistant strains. Pathog Dis. 2017;75(9). Epub 2017/12/01. doi: 10.1093/femspd/ftx118 . 29186408 10.1093/femspd/ftx118

[41] Lu LL, Chung AW, Rosebrock TR, Ghebremichael M, Yu WH, Grace PS, et al A Functional Role for Antibodies in Tuberculosis. Cell. 2016;167(2):433–43 e14. Epub 2016/09/27. doi: 10.1016/j.cell.2016.08.072 ; PubMed Central PMCID: PMCPMC5526202. 27667685 10.1016/j.cell.2016.08.072 PMC5526202

[42] Nimmerjahn F, Ravetch JV. The antiinflammatory activity of IgG: the intravenous IgG paradox. Journal of Experimental Medicine. 2007;204(1):11–5. doi: 10.1084/jem.20061788 17227911 10.1084/jem.20061788 PMC2118416

[43] Cymer F, Beck H, Rohde A, Reusch D. Therapeutic monoclonal antibody N-glycosylation–Structure, function and therapeutic potential. Biologicals. 2017. 10.1016/j.biologicals.2017.11.001 29239840

