# Supplementary material for: Adjunct antibody administration with standard treatment reduces relapse rates in a murine tuberculosis model of necrotic granulomas
Source: PLoS One. 2018 May 14;13(5):e0197474. doi: 10.1371/journal.pone.0197474 (PMC5951562; doi:10.1371/journal.pone.0197474)
Supplement: S1 Fig — Four to six-week-old female C3HeB/FeJ mice were aerosol infected with Mycobacterium tuberculosis. Mice were sacrificed one day after infection (to assess implantation) and at the start of treatment at six weeks after infection. Mice received multi-drug TB treatments with and without adjunctive treatments for 12 weeks and were split among six treatment groups: no treatment, standard TB treatment (administered rifampin, isoniazid and pyrazinamide by gavage), or standard TB treatment with either adjunctive etanercept (positive control), isotype antibody (negative control) or anti-MMP-9 antibody for 6 weeks. Pyrazinamide was administered for the first 8 weeks only, as is standard for TB treatment in humans. Additional cohorts of mice were held for 16 weeks after cessation of treatment to assess for stable, relapse free cure. (DOCX) [file pone.0197474.s001.docx]

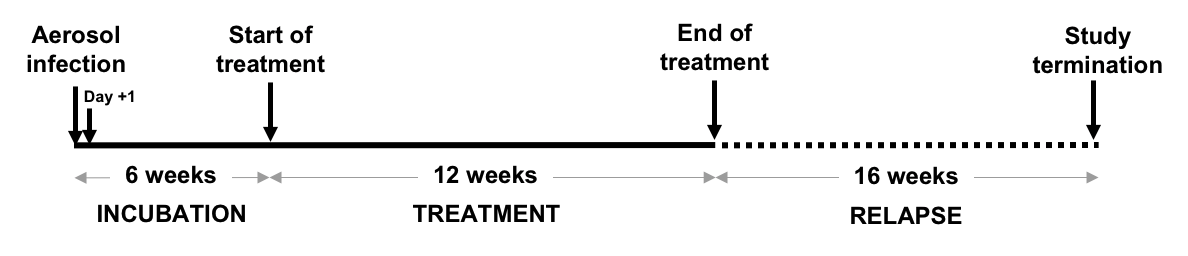


**S1 Fig. Experimental scheme.** Four to six-week-old female C3HeB/FeJ mice were aerosol infected with *Mycobacterium tuberculosis*. Mice were sacrificed one day after infection (to assess implantation) and at the start of treatment at six weeks after infection. Mice received multi-drug TB treatments with and without adjunctive treatments for 12 weeks and were split among six treatment groups: no treatment, standard TB treatment (administered rifampin, isoniazid and pyrazinamide by gavage), or standard TB treatment with either adjunctive etanercept (positive control), isotype antibody (negative control) or anti-MMP-9 antibody for 6 weeks. Pyrazinamide was administered for the first 8 weeks only, as is standard for TB treatment in humans. Additional cohorts of mice were held for 16 weeks after cessation of treatment to assess for stable, relapse free cure.
