# Supplementary material for: Adjunct antibody administration with standard treatment reduces relapse rates in a murine tuberculosis model of necrotic granulomas
Source: PLoS One. 2018 May 14;13(5):e0197474. doi: 10.1371/journal.pone.0197474 (PMC5951562; doi:10.1371/journal.pone.0197474)

**S2 Fig. Post-mortem gross pathological lungs samples.** Mice were sacrificed at 6 (A) and 8 (B) weeks of treatment and the lungs were harvested, fixed in 4% paraformaldehyde and gross images were acquired. RHZ = standard TB treatment comprising rifampin (R), isoniazid (H) and pyrazinamide (Z) administered by gavage.


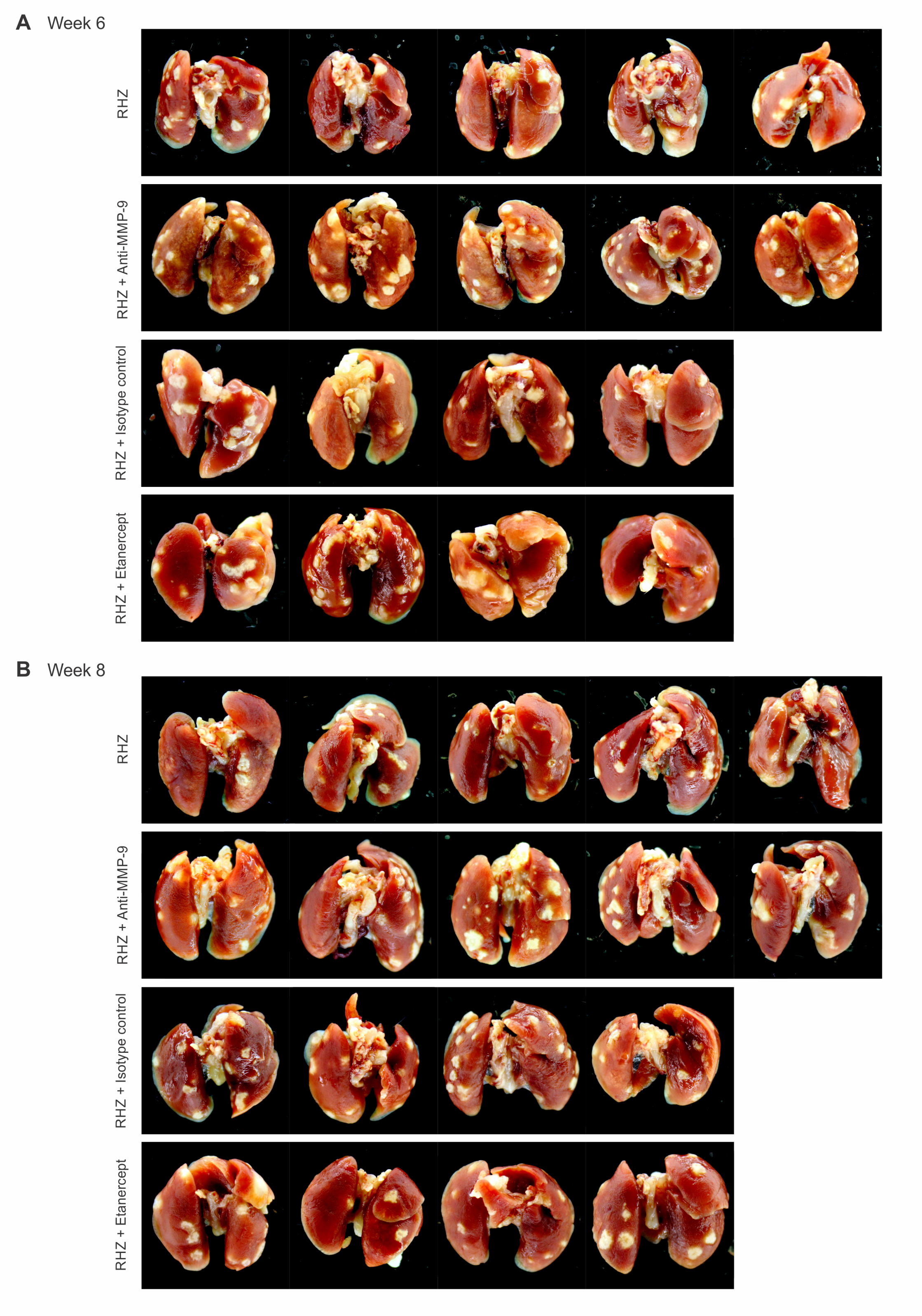

Supplement: S2 Fig — Mice were sacrificed at 6 (A) and 8 (B) weeks of treatment and the lungs were harvested, fixed in 4% paraformaldehyde and gross images were acquired. RHZ = standard TB treatment comprising rifampin (R), isoniazid (H) and pyrazinamide (Z) administered by gavage. (DOCX) [file pone.0197474.s002.docx]
