# Supplementary material for: Adjunct antibody administration with standard treatment reduces relapse rates in a murine tuberculosis model of necrotic granulomas
Source: PLoS One. 2018 May 14;13(5):e0197474. doi: 10.1371/journal.pone.0197474 (PMC5951562; doi:10.1371/journal.pone.0197474)
Supplement: S3 Fig — Additional cohorts of mice were held for 16 weeks after cessation of treatment to assess for stable, relapse free cure. 20 animals per group were CT scanned to evaluate for the presence of cavitary lesions. The transverse, coronal and sagittal views of a CT from a representative mouse scanned 16 weeks post-treatment are shown in panel A. The crosshairs indicate the cavitary lesion. A cavity was defined as a macroscopic region of air (density <−900 HU) within diseased lung parenchyma. (B) The number of cavitary lesions was quantified by CT in each of the scanned mice within the relapse groups. RHZ = standard TB treatment comprising rifampin (R), isoniazid (H) and pyrazinamide (Z) administered by gavage. (DOCX) [file pone.0197474.s003.docx]

**
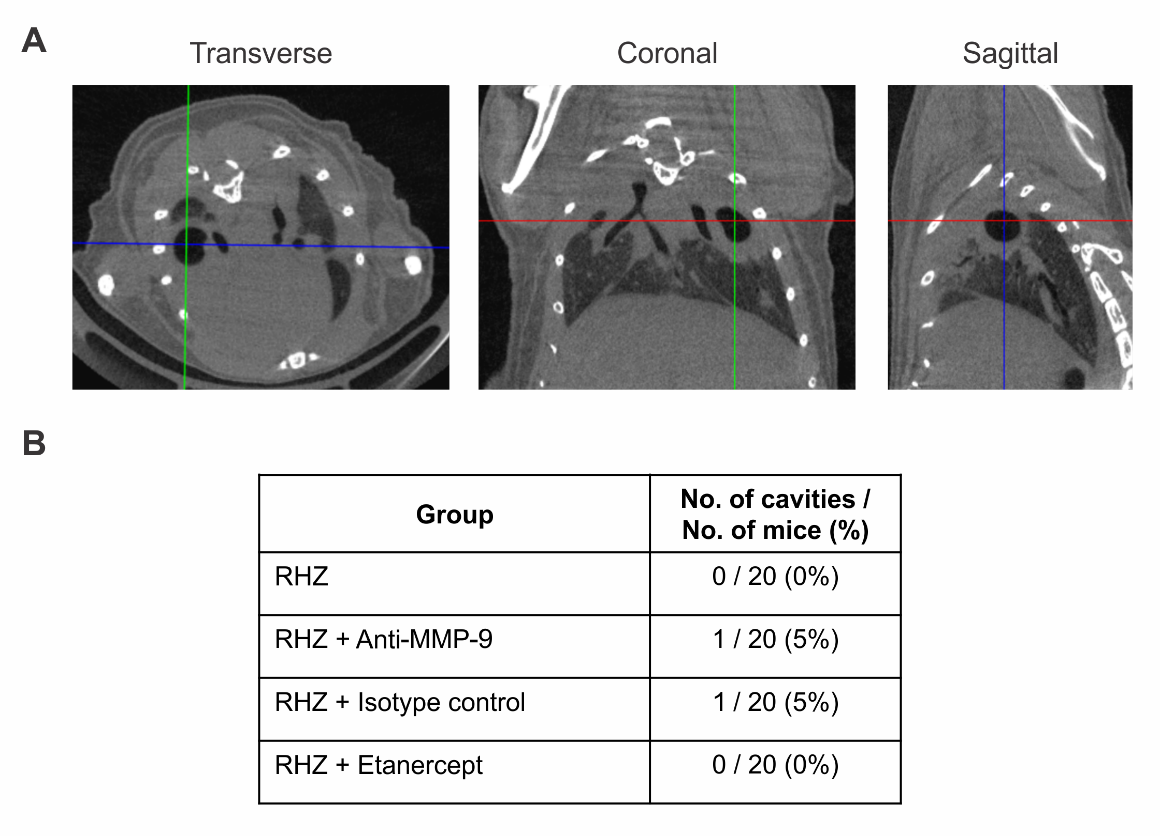
**

**S3 Fig. Presence of cavities in each treatment arm during relapse.** Additional cohorts of mice were held for 16 weeks after cessation of treatment to assess for stable, relapse free cure. 20 animals per group were CT scanned to evaluate for the presence of cavitary lesions. The transverse, coronal and sagittal views of a CT from a representative mouse scanned 16 weeks post-treatment are shown in panel A. The crosshairs indicate the cavitary lesion. A cavity was defined as a macroscopic region of air (density <−900 HU) within diseased lung parenchyma. (B) The number of cavitary lesions was quantified by CT in each of the scanned mice within the relapse groups. RHZ = standard TB treatment comprising rifampin (R), isoniazid (H) and pyrazinamide (Z) administered by gavage.
